# miR-614通过调控靶基因*PSA*表达抑制人肺癌细胞的侵袭和增殖能力

**DOI:** 10.3779/j.issn.1009-3419.2014.10.02

**Published:** 2014-10-20

**Authors:** 方 律, 奇 薛

**Affiliations:** 100021 北京，中国医学科学院肿瘤医院胸外科 Department of Thoracic Surgery, Cancer Institute and Hospital, Chinese Academy of Medical Sciences, Beijing 100021, China

**Keywords:** 肺肿瘤, MicroRNA, 嘌呤霉素敏感性氨肽酶, 侵袭, 增殖, Lung neoplasms, MicroRNA, Puromycin-sensitive aminopeptidase, Invasion, Proliferation

## Abstract

**背景与目的:**

MicroRNAs（miRNAs）是一种非编码的小分子RNA，在肿瘤的发生发展过程中发挥着重要的作用，各种miRNAs对肺癌的作用及机制仍需进一步阐明。本研究探讨了miR-614对肺癌细胞增殖和侵袭的作用及机制。

**方法:**

应用实时定量PCR检测miR-614在高低不同转移能力肺癌PGCL3和PGLH7细胞中的表达水平；脂质体2000介导分别转染miR-614类似物和抑制物入PGCL3和PGLH7细胞，应用Transwell实验检测miR-614对肺癌细胞侵袭的作用；应用CCK8实验和BrdU掺入实验检测miR-614对肺癌细胞增殖的作用；生物信息学软件预测miR-614潜在的靶基因，双荧光素酶报告基因验证miR-614是否作用于嘌呤霉素敏感性氨肽酶（puromycin-sensitive aminopeptidase, PSA）mRNA的3’UTR区预测靶位；Western blot检测PSA蛋白水平。

**结果:**

miR-614在高转移肺癌细胞PGCL3中的表达水平明显低于其在低转移肺癌细胞PGLH7的表达水平；体外侵袭实验结果显示，miR-614可抑制肺癌细胞侵袭；细胞增殖实验结果显示，miR-614可抑制肺癌细胞增殖；miRanda软件预测并经双荧光素酶报告基因验证PSA是miR-614的靶基因；miR-614类似物可下调PSA蛋白表达，miR-614抑制物可上调PSA蛋白表达。

**结论:**

miR-614过表达抑制了人肺癌细胞的侵袭和增殖能力，PSA是miR-614下游靶基因之一。

## Abstract (en)

**Background and objective:**

MicroRNAs (miRNAs) is a group of non-coding small RNA molecules, which play important roles in the development of tumor. The mechanisms of various kinds of miRNAs in lung cancer still need to be further elucidated. This study investigated the function of miR-614 on lung cancer cell invasion and proliferation.

**Methods:**

Real-time quantitative PCR was used to detect the expression of miR-614 in lung cancer cell PGCL3 and PGLH7. Transwell assay was used to test the role of miR-614 on regulating invasion and migration of cells. CCK8 assay and BrdU incorporation assay was used to assess the role of miR-614 on cell proliferation. Bioinformatics software predicted the potential target genes of miR-614 and dual luciferase reporter gene was used to analyze the binding between miR-614 and 3'UTR of puromycin-sensitive aminopeptidase (PSA). Western blot detected the PSA protein levels.

**Results:**

The expression of miR-614 in PGCL3 cells with high metastasis potential was significantly lower than that in PGLH7 cells with low metastasis potential. Furthermore, altered expression of miR-614 by transfection of pre-miR-614 mimics and inhibitor significantly affected the ability of invasion and proliferation of lung cancer cells. Bioinformatics analysis predicted that PSA was one of the potential target genes of miR-614. Altered expression of miR-614 markedly down-regulated the PSA protein levels of lung cancer cells. In addition, dual luciferase reporter gene assay indicated that miR-614 regulated PSA expression by binding to the 3'UTR of PSA mRNA.

**Conclusion:**

MiR-614 inhibited cell invasion and proliferationa targeting PSA in lung cancer cells, PGCL3.

肺癌是我国发病率和死亡率增长最快的恶性肿瘤，而造成患者死亡的主要原因是发生了远处转移。因此，有效地控制肺癌的侵袭转移已成为当前肺癌研究和治疗的重要课题和难题。微小RNA（microRNA, miRNA）是一类长约22 nt的非编码内源性小RNA，可以与基因mRNA的3’UTR区结合，起到在转录后水平调控基因表达的作用^[1, 2]^。

近年来，研究者们^[3-6]^发现miRNA在调控肿瘤分化、生长、浸润和转移等多个环节发挥重要作用。已有研究^[7]^发现多种miRNA可通过不同的途径调控肺癌细胞的侵袭转移，如miR-126、miR-183和let-7可分别通过作用于其各自的靶基因调控肺癌细胞的增殖和迁移能力^[8-12]^。因此，深入研究不同miRNAs家族成员对肺癌的作用对于阐明miRNAs在肺癌发生和发展中的机制具有重要意义。

在前期研究工作中，我们应用miRNA表达谱芯片检测了具有高低不同转移能力的肺癌细胞株PGCL3和PGLH7细胞，芯片结果显示miR-614在PGCL3中低表达。因此，在本研究中，我们应用实时定量PCR方法，验证miR-614在PGCL3和PGLH7细胞中的表达水平，并进一步探讨miR-614对肺癌细胞增殖和侵袭的作用及其可能机制，为后续基于miRNAs的肺癌临床生物治疗的新靶点开发提供实验依据。

## 1 材料和方法

### 1.1 试剂及细胞株

miRNA提取试剂盒（mirVana RNA Isolation Kit）、逆转录试剂盒（Taqman miRNA Reverse Transcripation Kit）、荧光实时定量PCR试剂盒（Taqman Universal PCR Master Mix No AmpErase UNG）和内参RNU6B MGB探针标记的引物均购于美国ABI公司。miR-614类似物Pre-miR-614 mimics、抑制剂Anti-miR-614 inhibitor、miR-614类似物阴性对照Pre-miR-614 mimics control和抑制剂阴性对照Anti-miR-614 inhibitor control均购于美国ABI公司。转染试剂Lipofectamine 2000购于Invitrogen公司。人源性肺巨细胞癌细胞株PGCL3和PGLH7细胞购于中国科学院上海生命科学研究院生物化学与细胞生物学研究所。RPMI-1640培养基购于Hyclone公司，胎牛血清购于Gibco公司。CCK-8试剂盒购于南京凯基生物有限公司。5-溴2-脱氧尿苷（BrdU）、PI购于Sigma公司，BrdU单克隆抗体购于NeoMarkers，FITC标记的羊抗鼠荧光二抗购于Sigma公司。Transwell小室购于Millipore公司。双荧光素酶报告基因检测试剂盒和pMIR-GLO载体购于美国Promega公司。PSA、β-actin抗体购于Santa Cruz公司，HRP标记的二抗购于Sigma公司。

### 1.2 细胞的培养和转染

人肺癌细胞株PGCL3和PGLH7细胞用含10%胎牛血清、1, 000 U/mL青霉素、100 mg/mL链霉素及2 mmol/L谷氨酰胺的RPMI-1640细胞培养液，于37 ℃、5%CO_2_条件下培养，待单层细胞生长达到80%融合时，用胰蛋白酶消化细胞，收集细胞，PBS冲洗1次后备用。将实验细胞分为miR-614类似物转染组、抑制物转染组、类似物阴性对照转染组、抑制物阴性对照转染组和只加转染试剂的空白对照组。细胞接种于6孔板内，待细胞约50%-60%融合时进行转染，每孔板加入25 pmol的类似物（抑制物及对照）和10 μL的转染试剂，使模拟物的终浓度为10 pmol/mL，转染后5 h换液。

### 1.3 miRNA的实时定量PCR检测

收获的细胞样品按照ABI公司miRNA提取试剂盒、逆转录试剂盒和荧光实时定量PCR试剂盒中说明书的方法进行操作，以RNU6B作为内参进行相对定量，每组设置3个复孔，在ABI 7500实时定量荧光PCR仪机器检测。

### 1.4 Transwell侵袭实验

将含10%血清的RPMI-1640培养基600 μL加入Transwell的下室，在有预置Matrigel的上室中按照不同实验分组分别加入经消化处理的细胞不含血清的RPMI-1640稀释的细胞悬液100 μL，置37 ℃、5%CO_2_细胞培养箱培养24 h后取出小室，用棉签擦去上层细胞，用2.5%戊二醛固定15 min，并以0.5% Triton X-100处理3 min，苏木素染核15 min。将培养小室倒置，在荧光倒置显微镜下观察、照相，并每组随机选取5个视野（200×）计数，取平均细胞数。

### 1.5 CCK-8方法检测细胞增殖能力

将细胞接种在96孔板内，每孔接种5, 000个细胞，分别在转染1 d-6 d内检测。100 μL的RPMI-1640培养液中加入10 μL的CCK8检测试剂，在细胞培养箱中继续培养2 h后，在酶标仪450 nm处检测各孔OD值，每组重复4次。相对增殖活力=处理组OD值/空白对照组OD值。

### 1.6 BrdU掺入实验

将细胞接种在24孔平板中，转染后换液继续培养，当细胞生长至80%汇合时在细胞中加10 μmol/L的BrdU，37 ℃下温育4 h。弃去细胞培养液，PBS冲洗3次，每次5 min。加入70%无水乙醇，4 ℃条件下固定10 min。弃去70%无水乙醇，PBS冲洗3次，每次5 min。加入2 mol/L的HCl，37 ℃下放置40 min，使细胞DNA变性。弃去HCl，PBS冲洗3次，每次10 min。在细胞上加入1.0%的BSA，在室温条件下放置1 h，进行封闭。PBS冲洗3次，每次5 min。吸干细胞表面的PBS，加入BrdU单克隆抗体（1:300稀释，100 μL/孔），4 ℃条件下孵育过夜。次日加入FITC标记的羊抗鼠荧光二抗，在室温条件下孵育2 h。用PI复染所有细胞的细胞核后，在荧光显微镜下进行观察。随机选择10个非重叠视野（×100）计算BrdU阳性细胞数，计算平均值。

### 1.7 生物信息学软件靶基因预测

应用在线生物信息学miRNA靶基因预测软件miRanda（http://www.miRBase.org/）对miR-614的靶基因进行预测。

### 1.8 双荧光素酶报告基因检测

克隆扩增*PSA*基因的3’UTR区的全长，引物序列：Forward primer 5’-CACGAGCTCTAATAAGGAAACATCTTTCATAGCC-3’，Reverse primer 5’-TTTCTCGAGTTCACATTGACATTTTTATTAACGC-3’，PCR产物克隆到pMIR-GLO（Promega）Luciferase基因下游的多克隆位点中，并针对生物信息学预测miR-614与嘌呤霉素敏感性氨肽酶基因的靶结合位点进行定点突变，表达海肾荧光素酶的pRL-TK载体用来作为内参照调整细胞数量和转染效率的差异，miR-614类似物以及阴性对照分别和荧光素酶报告载体共转染进入PGCL3细胞，按照Promega提供的方法进行双荧光素酶活性检测^[13]^。

### 1.9 Western blot

收集各实验组转染后细胞，细胞裂解液处理细胞后加入适量电泳上样缓冲液，沸水中加热10 min，在12%聚丙烯酰胺凝胶中进行电泳，转膜1 h至PVDF膜上，3%BSA在37 ℃条件下封闭2 h，分别加入鼠抗人PSA抗体（1:500）、鼠抗人β-actin抗体（1:500）4 ℃过夜，PBST洗涤3次，每次10 min：加HRP标记的相应二抗（1:4, 000），37 ℃培育30 min，PBST洗3次，每次15 min。用化学发光试剂对膜进行处理，于暗室中曝光、显影与定影，并拍片记录。

### 1.10 统计学方法

采用SPSS 13.0统计软件分析，数据结果以Mean±SD的方式显示，多组间均数比较采用单因素方差分析（*ANOVA*），*P* < 0.05为差异具有统计学意义。

## 2 结果

### 2.1 miR-614在高转移能力肺癌细胞中表达降低

实时定量PCR检测高转移能力肺癌细胞PGCL3和低转移能力肺癌细胞PGLH7细胞中miR-614的表达水平，结果显示，PGLH7细胞中miR-614的表达水平比PGCL3细胞中表达水平高（6.95±0.21）倍（*P* < 0.01），提示miR-614在肺癌细胞中的表达降低可能与肺癌细胞的高转移能力相关。

### 2.2 miR-614表达异常改变了肺癌细胞的侵袭能力

为了进一步证实miR-614能抑制肺癌细胞的转移，我们用Transwell小室跨膜迁移实验检测了miR-614表达的异常对肺癌细胞侵袭运动能力的改变。在PGCL3细胞中分别转染miR-614 mimics和miR-614 mimics control，在PGLH7细胞中分别转染miR-614 inhibitor和miR-614 inhibitor control。与对照组相比，转染miR-614 mimics增加PGCL3细胞miR-614表达水平后，细胞侵袭运动能力明显降低（*P* < 0.01）；与之对应，转染miR-614 inhibitor降低PGLH7细胞miR-614表达水平后，细胞侵袭运动能力明显提高（*P* < 0.01）（图 1）。该结果表明，miR-614可抑制肺癌细胞的侵袭能力。

**1:**
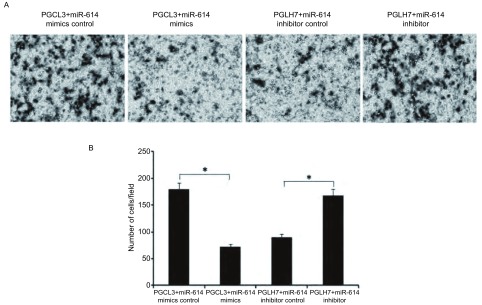
miR-614对人肺癌细胞体外侵袭能力的影响。A：在PGCL3细胞中分别转染miR-614 mimics和miR-614 mimics control，在PGLH7细胞中分别转染miR-614 inhibitor和miR-614 inhibitor control，应用Transwell实验检测miR-614对肺癌细胞侵袭能力的作用；B：直方图显示侵润的细胞数发生明显变化，*P < 0.01。 The effects of miR-614 on the invasion of human lung cancer cell lines *in vitro*. A: Lung cancer PGCL3 cells were transfected with miR-614 mimics and miR-614 mimics control and PGLH7 cells with miR-614 inhibitor and miR-614 inhibitor control and then subjected to tumor cell Transwell invasion assay; B: The histogram showed that the number of invasive cell was significantly changed. *P < 0.01.

### 2.3 miR-614表达异常改变了细胞的增殖能力

我们进一步检测了miR-614改变异常是否能够影响细胞的增殖能力。在PGCL3细胞中分别转染miR-614 mimics和miR-614 mimics control，在PGLH7细胞中分别转染miR-614 inhibitor和miR-614 inhibitor control。用CCK-8方法检测细胞活力绘制增殖曲线，结果表明，转染miR-614 inhibitor降低PGLH7细胞miR-614水平，与未处理组及阴性对照组相比，细胞增殖能力明显增强（*P* < 0.01）（图 2A）；与之对应，转染miR-614 mimics增加PGCL3细胞中miR-614的水平后，与未处理组及阴性对照组相比，细胞增殖能力明显降低（*P* < 0.01）（图 2B）。我们又利用BrdU掺入实验检测了miR-614表达改变对细胞增殖能力的影响。结果如图 3所示，转染miR-614 mimics增加PGCL3细胞miR-614水平后，细胞BrdU掺入明显减少；与之对应，转染miR-614 inhibitor降低PGLH7细胞miR-614水平后，细胞BrdU掺入明显增加（*P* < 0.01）。这些结果表明，miR-614可抑制肺癌细胞的增殖。

**2:**
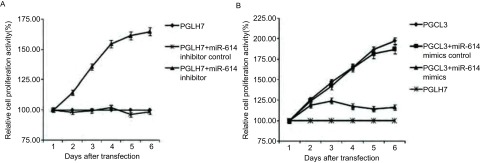
CCK-8实验检测细胞增殖能力。A：在PGLH7细胞中分别转染miR-614 inhibitor和miR-614 inhibitor control，应用CCK-8实验检测细胞活力；B：在PGCL3细胞中分别转染miR-614 mimics和miR-614 mimics control，应用CCK-8实验检测细胞活力。 The cell proliferation assay by CCK-8 kit. A: Lung cancer PGLH7 cells were transfected with miR-614 inhibitor and miR-614 inhibitor control for CCK-8 kit detection of cell viability; B: Lung cancer PGCL3 cells were transfected with miR-614 mimics and miR-614 mimics control for CCK-8 kit detection of cell viability.

**3:**
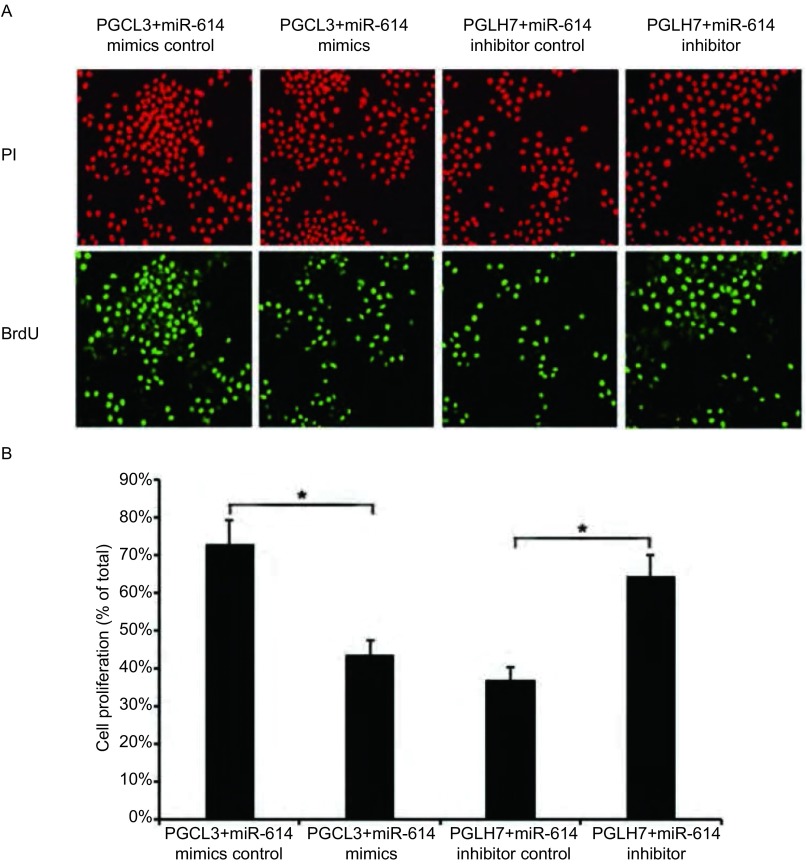
BrdU掺入实验检测细胞增殖能力。A：在肺癌细胞中分别转染miR-614 mimics、miR-614 mimics control、miR-614 inhibitor和miR-614 inhibitor control，应用BrdU掺入实验检测细胞增殖能力；B：直方图显示细胞增殖能力发生明显变化，*P < 0.01。 The cell proliferation determined by BrdU assay. A: Lung cancer cells were transfected with miR-614 mimics control, miR-614 mimics, miR-614 inhibitor control, or miR-614 inhibitor and subjected to BrdU incorporation assay; B: The histogram showed that the cell proliferation was significantly changed. *P < 0.01.

### 2.4 嘌呤霉素敏感性氨肽酶是miR-614调节的下游靶基因

为了进一步确定miR-614在肺癌细胞中调节的下游靶基因，我们利用在线生物信息学软件miRanda预测发现嘌呤霉素敏感性氨肽酶的mRNA的3’UTR含有能和miR-614成熟序列结合的位点，提示PSA可能是miR-614的靶基因（图 4A）。为了进一步证实miR-614对肺癌细胞PSA表达的调节，我们检测了肺癌细胞中miR-614和PSA表达水平之间的关系。在PGCL3细胞中转染miR-614 mimics增加miR-614的表达水平后，与miR-614 mimics control组相比PSA蛋白表达明显降低；在PGLH7细胞中转染miR-614 inhibitor降低miR-614的表达水平后，与miR-614 inhibitor control组相比PSA蛋白表达明显增加（图 4B）。这一结果表明miR-614抑制了肺癌细胞中PSA的表达。为了进一步明确miR-614通过与3’UTR结合调节PSA表达，我们构建了含有PSA的3’UTR序列的荧光素酶报告基因表达载体WT-PSA，同时构建了PSA 3’UTR区miR-614结合种子序列突变的载体Mut-PSA。PGCL3细胞中，表达野生型PSA的3’UTR序列的细胞中，转染miR-614 mimics上调miR-614的水平与转染miRNA对照组相比荧光素酶活性明显下降（*P* < 0.01），说明miR-614抑制了荧光素酶的表达。转染突变型PSA的3’UTR序列的细胞中，miR-614 mimics上调miR-614的水平与对照组相比荧光素酶活性无明显差异，说明与miR-614是通过打靶并结合PSA的3’UTR来调节蛋白表达的（图 4C）。

**4:**
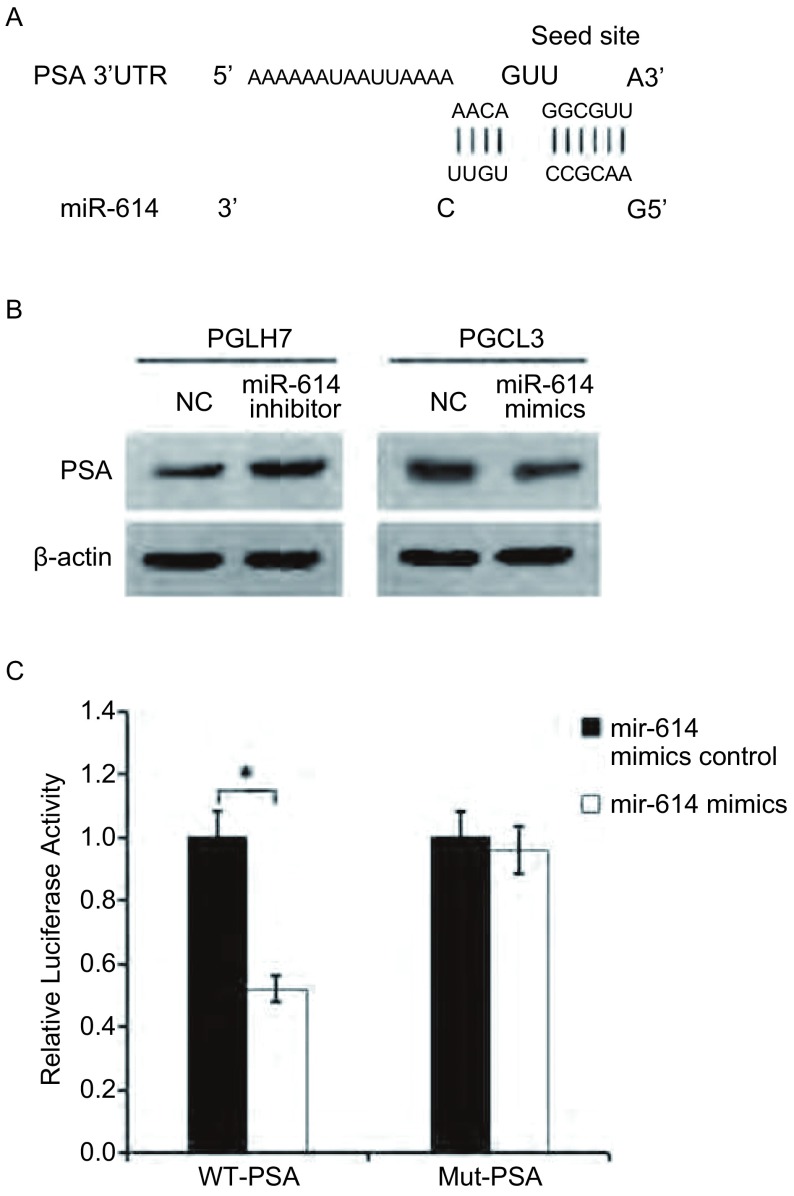
*PSA*是miR-614的靶基因。A：miRanda在线生物信息学软件预测miR-614靶基因。B：miR-614对人肺癌细胞中PSA蛋白表达水平的影响。C：双荧光素酶报告基因检测结果。*P < 0.01。 *PSA* as miR-614 targeting gene. A: Potential targeting gene of miR-614 predicted by miRanda online bioinformatics software; B: The effect of miR-614 on the protein expression of PSA on human lung cancer cell lines; C: Dual-luciferase reporter gene assay; *P < 0.01. PSA: puromycin-sensitive aminopeptidase.

## 3 讨论

肺癌是我国发病率和死亡率增长最快的恶性肿瘤，而造成患者死亡的主要原因是肺癌很早就发生了远处转移。因此，有效地控制肺癌的侵袭转移已成为当前肺癌研究和治疗的重要课题和难题。肿瘤侵袭转移是一个多步骤的过程，是各种粘附分子、蛋白水解酶类、细胞因子和血管生成因子等多种因素共同参与调节的复杂过程。MicroRNA（miRNA）是近年来新发现的一类长度为18 nt-25 nt进化上比较保守的、非编码蛋白质的小分子RNA，它与靶基因mRNA 3’UTR区通过不完全配对结合抑制靶基因的转录后调控，这类小分子物质参与了细胞增殖、分化、凋亡等多种重要细胞活动的调控^[14, 15]^。近年来大量研究^[16, 17]^表明，miRNAs与人类多种肿瘤的发生、发展及侵袭转移存在着密切关系。目前，与肺癌相关的miRNA研究也取得了一些进展，但有哪些miRNA对肺癌发生发展具有调控作用，其机制如何等等很多问题仍是未知的，还需要我们进行大量全面、深入的研究工作。

在前期研究工作中，我们对具有高低不同转移潜能的肺癌细胞株PGCL3和PGLH7细胞的miRNA表达谱进行了分析，筛选到了一些与肺癌细胞转移密切相关的miRNA，其中miR-614在PGCL3细胞中的表达水平明显低于其在PGLH7细胞中表达水平，提示miR-614在肺癌细胞中的低表达可能与肺癌细胞的高转移能力相关。在此基础上，本研究中我们进一步应用real-time PCR方法在PGCL3和PGLH7细胞中验证了miR-614的表达水平，其在高转移肺癌细胞PGCL3中的表达水平明显低于低转移肺癌细胞PGLH7，与芯片检测结果相吻合，提示miR-614高表达可能抑制肺癌细胞的侵袭。目前在国内外研究中，还未见miR-614与肺癌转移关系的相关报道。为了研究miR-614是否影响肺癌细胞的侵袭转移能力，在本研究中，我们进一步应用Transwell实验方法研究了miR-614对肺癌细胞侵袭的影响。实验结果显示，PGCL3细胞转染miR-614 mimics后细胞的穿膜能力明显降低；PGLH7细胞转染miR-614 inhibitor后细胞的穿膜能力明显提高。表明miR-614可抑制肺癌细胞的侵袭。

为了全面了解miR-614对肺癌细胞生物学功能的调节作用，我们进一步应用CCK8试剂盒和BrdU掺入方法研究了miR-614对肺癌细胞增殖的影响。Cell Counting Kit-8简称CCK-8试剂盒，是一种基于WST-8的广泛应用于细胞增殖和细胞毒性的快速高灵敏度检测试剂盒。BrdU掺入方法也是一种很好的分析细胞周期的方法，并且其应用范围越来越广泛。5-溴-2-脱氧尿苷（5-bromo-2-deoxyuridine, BrdU）为DNA前体胸腺嘧啶核苷的类似物，在细胞增殖过程中，能选择性的掺入到处于细胞周期S期（即DNA合成期）细胞的单链DNA核苷酸序列中。在活体中引用可示踪DNA合成的前体物质BrdU，使BrdU竞争性的替代胸腺嘧啶而掺入到增殖期的细胞中，成为细胞增殖的重要标志。掺入合成的BrdU的强度可直接显示细胞增殖的能力，再取其相应组织制作切片，应用与BrdU特异反应的单克隆抗体，对已完成了BrdU标记的正常或病理状态下细胞的DNA复制进行检测，并可进行定位观察和统计学分析。我们研究发现，CCK-8和BrdU掺入方法实验结果均显示细胞转染miR-614 mimics后增殖能力减弱，转染miR-614 inhibitor后增殖能力增强，表明miR-614可抑制肺癌细胞的增殖作用。

为了进一步探讨miR-614抑制肺癌细胞侵袭和增殖的作用机制，我们应用在线生物信息学miRNA靶基因预测软件miRanda对miR-614的靶基因进行了预测，软件预测PSA可能为miR-614的靶基因。为了证明miR-614和PSA基因之间的调控关系，我们对肺癌细胞株进行miR-614 mimics和inhibitor的转染分别促进和抑制miR-614的表达，并应用双荧光素酶报告基因检测系统和Western印迹方法从基因和蛋白两个水平验证两者之间的靶向调控关系，证明了miR-614可以直接调控*PSA*基因。目前，未见PSA在肺癌中作用的相关文献报道，在后续研究中，我们将对PSA在肺癌细胞中发挥的生物功能进行深入研究。并将进一步研究在肺癌细胞中*PSA*作为miR-614的靶基因是否介导了miR-614对细胞侵袭和增殖能力的调节。

综上所述，我们的研究表明肺癌细胞中miR-614的高表达抑制了细胞侵袭和增殖能力，*PSA*基因是miR-614调控的下游靶基因。在后续研究中，我们将继续深入探讨miR-614在肺癌细胞生长和转移中的作用及可能机制，以期为肺癌发生和转移机制的研究以及临床肺癌生物治疗提供新的靶点。
